# Supplementary material for: Expanding the genome-targeting scope and the site selectivity of high-precision base editors
Source: Nat Commun. 2020 Jan 31;11:629. doi: 10.1038/s41467-020-14465-z (PMC6994485; doi:10.1038/s41467-020-14465-z)
Supplement: Supplementary file 1 — Supplemenatary Information [file 41467_2020_14465_MOESM1_ESM.pdf]

# Supplementary Information

Expanding the genome-targeting scope and the site selectivity of high-precision base editors

Tan et al.

**Supplementary Table 1** Target protospacer sequences analyzed in this study. Target Cs are shown in red, with the subscript numbers indicating their position relative to the PAM. The PAM sequence is shown in blue.

| Target | Sequence (5' → 3') | Analysis method |
|---|---|---|
| Can1-1 | ATACTAAT$C_{-12}C_{-11}$ATGCCGCCAGTGG | canavanine selection |
| Can1-2 | GCAAATT$C_{-13}$AAATATTTACGTTGG | canavanine selection |
| Can1-3 | ACGT$C_{-16}C_{-15}$AAAATTGAATGACTTGG | canavanine selection |
| Can1-4 | TTT$C_{-17}$AAGGTACTGAACTAGTTGG | canavanine selection |
| Can1-5 | T$C_{-19}C_{-18}$AATAACGGAATCCAACTGGG | canavanine selection |
| PolyC-1-NGA | A$C_{-19}C_{-18}C_{-17}C_{-16}C_{-15}C_{-14}C_{-13}C_{-12}$TCATCTTTGAGTGA | NGS |
| PolyC-2-NGA | $C_{-20}C_{-19}C_{-18}C_{-17}C_{-16}C_{-15}C_{-14}$TGAGGCTTATGAGAGA | NGS |
| PolyC-3-NGCG | T$C_{-19}C_{-18}C_{-17}C_{-16}C_{-15}C_{-14}$TTCTGCCCAATTAGGCG | NGS |
| PolyC-4-NGCG | TT$C_{-18}C_{-17}C_{-16}C_{-15}C_{-14}C_{-13}$ACTCACAGGAAGAGCG | NGS |
| PolyC-5-NGC | A$C_{-19}C_{-18}C_{-17}C_{-16}C_{-15}$AA$C_{-12}C_{-11}C_{-10}C_{-9}C_{-8}C_{-7}C_{-6}$ATCAGTGC | NGS |
| PolyC-6-NGT | $C_{-20}C_{-19}C_{-18}C_{-17}C_{-16}C_{-15}C_{-14}C_{-13}C_{-12}$TTGATACTTCCTGT | NGS |
| PolyC-7 | $C_{-21}C_{-20}C_{-19}C_{-18}C_{-17}C_{-16}C_{-15}C_{-14}C_{-13}$ATGTT$C_{-7}C_{-6}$GA GATCGG | NGS |
| PolyC-8 | TATA$C_{-20}C_{-19}C_{-18}C_{-17}C_{-16}C_{-15}C_{-14}C_{-13}$TATATGGTAAAAAGG | NGS |
| PSEN1-L166P | GC$C_{-18}$TATTATATCATCTCTATTGT | - |
| TYR-Y327C | ATTCA$C_{-15}$ATTGGGTCAAACTCAGG | - |

**Supplementary Table 2** Width of the editing windows of A3A-derived BEs in two polycytidine motifs (polyC-7 and polyC-8; cf. Fig. 2). The approximate width of the editing window was defined as the number of nucleotides within which editing efficiency exceeds the half-maximal value (21).

| Base editor | PolyC-7 | PolyC-8 |
|---|---|---|
| A3A-BE3 | 5 | 6 |
| A3A-NL-BE3 | 9 | 7 |
| A3AΔ194-BE3 | 4 | 4 |
| A3AΔ190-BE3 | 6 | 3 |
| A3AΔ186-BE3 | 4 | 2 |
| A3AΔ182-BE3 | 3 | 2 |
| A3AΔ178-BE3 | 3 | 2 |

**Supplementary Table 3** Oligonucleotides used in this study.

***Primers for construction of sgRNA plasmids***

| Primer name | Sequence (5' → 3') |
|---|---|
| sgRNA-Can1-1 | AAAGATAAATGATCGATACTAATCCATGCCGCCAGGTTTTAGAGCTAGAAATAGCAAGT |
| sgRNA-Can1-2 | AAAGATAAATGATCGGCAAATTCAAATATTTACGTGTTTTAGAGCTAGAAATAGCAAGT |
| sgRNA-Can1-3 | AAAGATAAATGATCGACGTCCAAAATTGAATGACTGTTTTAGAGCTAGAAATAGCAAGT |
| sgRNA-Can1-4 | AAAGATAAATGATCGTTTCAAGGTACTGAACTAGTGTTTTAGAGCTAGAAATAGCAAGT |
| sgRNA-Can1-5 | AAAGATAAATGATCGTCCAATAACGGAATCCAACTGTTTTAGAGCTAGAAATAGCAAGT |
| sgRNA-PolyC-1 | AAAGATAAATGATCGACCCCCCCCTCATCTTTGAGGTTTTAGAGCTAGAAATAGCAAGT |
| sgRNA- PolyC-2 | AAAGATAAATGATCGCCCCCCCCTGAGGCTTATGAGGTTTTAGAGCTAGAAATAGCAAGT |
| sgRNA- PolyC-3 | AAAGATAAATGATCGTCCCCCCTTCTGCCCAATTAGTTTTAGAGCTAGAAATAGCAAGT |
| sgRNA- PolyC-4 | AAAGATAAATGATCGTTCCCCCCACTCACAGGAAGGTTTTAGAGCTAGAAATAGCAAGT |
| sgRNA- PolyC-5 | AAAGATAAATGATCGACCCCCAACCCCCCCATCAGGTTTTAGAGCTAGAAATAGCAAGT |
| sgRNA- PolyC-6 | AAAGATAAATGATCGCCCCCCCCCCTTGATACTTCCGTTTTAGAGCTAGAAATAGCAAGT |
| sgRNA- PolyC-7 | AAAGATAAATGATCGCCCCCCCCCATGTTCCGAGATGTTTTAGAGCTAGAAATAGCAAGT |
| sgRNA- PolyC-8 | AAAGATAAATGATCGCCCCCCCCCTATATGGTAAAAGTTTTAGAGCTAGAAATAGCAAGT |
| sgRNA-Rev | TATAGGGCGAATTGGGTACCGGCCGCAAATTAAAG |

***Primers for construction of base editors***

| Primer name | Sequence (5' → 3') | Constructs |
|---|---|---|
| VQR-BE3-1F | GGACAAGGGTAGGGATTTCG | VQR-BE3 |
| VQR-BE3-1R | GAATCCGCCGTATTTCTTGG | |
| VQR-BE3-2F | CCAAGAAATACGGCGGATTCGTTTCTCCTACAGTCGCTTACAG | |
| VQR-BE3-2R | CAGGACCTCCTTTGTAGATCTGTATTGCTTTCTGTCTATGGTGGTGT | |
| VQR-BE3-3F | GATCTACAAAGGAGGTCCTG | |
| VQR-BE3-3R | TTGAATAACTAAAGCCCATG | |
| VRER-BE3-1F | GGACAAGGGTAGGGATTTCG | VRER-BE3 |
| VRER-BE3-1R | ACCTTTCTGCAGCTCGCGCGCACTAGCGAGCAT | |
| VRER-BE3-2F | GAGCTGCAGAAAGGTAACGA | |
| VRER-BE3-2R | CTTTGTAGATCTGTATTCCTTTCTGTCTATGGT | |
| VRER-BE3-3F | TACAGATCTACAAAGGAGGT | |
| VRER-BE3-3R | ATGTTACATGCGTACACGCG | |

| | | |
|---|---|---|
| Cas9NG-BE3-1F | GGACAAGGGTAGGGATTTCG | Cas9-NG-BE3 |
| Cas9NG-BE3-1R | GCTGTTCCTTTTCGGGCGGATACTTTCCTTGGA | |
| Cas9NG-BE3-2F | CCGAAAAGGAACAGCGACAA | |
| Cas9NG-BE3-2R | GTTACCTTTCTGCAGGAAGCGCGCACTAGCGAGCAT | |
| Cas9NG-BE3-3F | CTGCAGAAAGGTAACGAGCT | |
| Cas9NG-BE3-3R | AAGTACTTGAAGGCTCGAGGCGCGCCCAAGTT | |
| Cas9NG-BE3-4F | GCCTTCAAGTACTTCGACACCACCATAGACAGAAAGGTATACAGATCTACAAAGGAGG | |
| Cas9NG-BE3-4R | ATGTTACATGCGTACACGCG | |
| cCDA1v-1F | GGACAAGGGTAGGGATTTCG | cCDA1-VQR/VRER/NG-BE3 |
| cCDA1v-1R | CAGATTCAGAAGTACCTGGAGTTTCAGAACCAGAGTCTCCACCGAGCTGAGAGA | |
| cCDA1v-2F | TCCAGGTACTTCTGAATCTGCTACTCCAGAATCTATGACCGACGCTGAGTACGTGAG | |
| cCDA1v-2R | ATTTGCAGGCATTTGCTCGGCATGCCGGTAGAGGTGTGGT | |
| A3A-NL-1F | AGAAAAAAACCCCGGATTCTAGAACTAGTGGATCCCCCGGGAAAAAAATGGAAGCCAGCCCAGCATC | A3A-NL-BE3 |
| A3A-NL-1R | TCTTGTCCATGTTTCCCTGATTCTGGAGAA | |
| A3A-NL-2F | TCAGGGAAACATGGACAAGAAGTACTCCAT | |
| A3A-NL-2R | ATTACTAAAGATCTCCTGCAGGTAGCAGATCCGATTCTTTC | |
| A3B-NL-1F | AGAAAAAAACCCCGGATTCTAGAACTAGTGGATCCCCCGGGAAAAAAATGAATCCACAGATCAGAAA | A3B-NL-BE3 |
| A3B-NL-1R | TCTTGTCCATGTTTCCCTGATTCTGGAGAA | |
| A3B-NL-2F | TCAGGGAAACATGGACAAGAAGTACTCCAT | |
| A3B-NL-2R | ATTACTAAAGATCTCCTGCAGGTAGCAGATCCGATTCTTTC | |
| hAID-NL-1F | AGAAAAAAACCCCGGATTCTAGAACTAGTGGATCCCCCGGGAAAAAAATGGACAGCCTCTTGATGAA | hAID-NL-BE3 |
| hAID -NL-1R | TCTTGTCCATAAGTCCCAAAGTACGAAATG | |
| hAID -NL-2F | TTTGGGACTTATGGACAAGAAGTACTCCAT | |
| hAID -NL-2R | ATTACTAAAGATCTCCTGCAGGTAGCAGATCCGATTCTTTC | |
| mAID-NL-1F | AGAAAAAAACCCCGGATTCTAGAACTAGTGGATCCCCCGGGAAAAAAATGGACAGCCTTCTGATGAA | mAID-NL-BE3 |
| mAID -NL-1R | TCTTGTCCATAAATCCCAACATACGAAATG | |
| mAID -NL-2F | GTTGGGATTTATGGACAAGAAGTACTCCAT | |

| | | |
|---|---|---|
| mAID -NL-2R | ATTACTAAAGATCTCCTGCAGGTAGCAGATCCGATTCTTTC | |
| cAICDA -NL-1F | AGAAAAAAACCCCGGATTCTAGAACTAGTGGATCCCCCGGGAAAAAAATGAGCAAGCTGGACAGTGT | cAICDA-NL-BE3 |
| cAICDA -NL-1R | TCTTGTCCATAAGGCCCAGCAGAGCGAAGC | |
| cAICDA -NL-2F | GCTGGGCCTTATGGACAAGAAGTACTCCAT | |
| cAICDA -NL-2R | ATTACTAAAGATCTCCTGCAGGTAGCAGATCCGATTCTTTC | |
| A3G -NL-1F | AGAAAAAAACCCCGGATTCTAGAACTAGTGGATCCCCCGGGAAAAAAATGAAACCGCATTTTCGCAA | A3G-NL-BE3 |
| A3G -NL-1R | TCTTGTCCATATTTTCCTGGTTTTGCAGGA | |
| A3G -NL-2F | CCAGGAAAATATGGACAAGAAGTACTCCAT | |
| A3G -NL-2R | ATTACTAAAGATCTCCTGCAGGTAGCAGATCCGATTCTTTC | |
| A3A-1F | AGAAAAAAACCCCGGATTCTAGAACTAGTGGATCCCCCGGGAAAAAAATGGAAGCCAGCCCAGCATC | A3A-BE3 |
| A3A -1R | CAGATTCAGAAGTACCTGGAGTTTCAGAACCAGAGTTTCCCTGATTCTGGAGAA | |
| A3A-2F | TCCAGGTACTTCTGAATCTGCTACTCCAGAATCTATGGACAAGAAGTACTCC | |
| A3A-2R | ATTACTAAAGATCTCCTGCAGGTAGCAGATCCGATTCTTTC | |
| A3A194-1F | AGAAAAAAACCCCGGATTCTAGAACTAGTGGATCCCCCGGGAAAAAAATGGAAGCCAGCCCAGCATC | A3AΔ194-BE3 |
| A3A194 -1R | TCTTGTCCATGAGAATGGCCCGCAGCCTCC | |
| A3A194-2F | GGCCATTCTCATGGACAAGAAGTACTCCAT | |
| A3A194-2R | ATTACTAAAGATCTCCTGCAGGTAGCAGATCCGATTCTTTC | |
| A3A190-1F | AGAAAAAAACCCCGGATTCTAGAACTAGTGGATCCCCCGGGAAAAAAATGGAAGCCAGCCCAGCATC | A3AΔ190-BE3 |
| A3A190 -1R | TCTTGTCCATCAGCCTCCCACTCAGGGCTT | |
| A3A190-2F | TGGGAGGCTGATGGACAAGAAGTACTCCAT | |
| A3A190-2R | ATTACTAAAGATCTCCTGCAGGTAGCAGATCCGATTCTTTC | |
| A3A186-1F | AGAAAAAAACCCCGGATTCTAGAACTAGTGGATCCCCCGGGAAAAAAATGGAAGCCAGCCCAGCATC | A3AΔ186-BE3 |
| A3A186 -1R | TCTTGTCCATCAGGGCTTGGCTGTGCTCAT | |
| A3A186-2F | CCAAGCCCTGATGGACAAGAAGTACTCCAT | |
| A3A186-2R | ATTACTAAAGATCTCCTGCAGGTAGCAGATCCGATTCTTTC | |
| A3A182-1F | AGAAAAAAACCCCGGATTCTAGAACTAGTGGATCCCCCGGGAAAAAAATGGAAGCCAGCCCAGCATC | A3AΔ182-BE3 |
| A3A182 -1R | TCTTGTCCATGTGCTCATCTAGTCCATCCC | |

| | | |
|---|---|---|
| A3A182-2F | AGATGAGCACATGGACAAGAAGTACTCCAT | |
| A3A182-2R | ATTACTAAAGATCTCCTGCAGGTAGCAGATCCGATTCTTTC | |
| A3A178-1F | AGAAAAAACCCCGGATTCTAGAACTAGTGGATCCCCCGGGAAAAAAATGGAAGCCAGCCCAGCATC | A3AΔ178-BE3 |
| A3A178 -1R | TCTTGTCCATTCCATCCCAGGGCTGGAAGG | |
| A3A178-2F | CTGGGATGGAATGGACAAGAAGTACTCCAT | |
| A3A178-2R | ATTACTAAAGATCTCCTGCAGGTAGCAGATCCGATTCTTTC | |
| A3A154-1F | AGAAAAAACCCCGGATTCTAGAACTAGTGGATCCCCCGGGAAAAAAATGGAAGCCAGCCCAGCATC | A3AΔ154-BE3 |
| A3A154 -1R | TCTTGTCCATGGTCATGATGGAGACTTGGG | |
| A3A154-2F | CATCATGACCATGGACAAGAAGTACTCCAT | |
| A3A154-2R | ATTACTAAAGATCTCCTGCAGGTAGCAGATCCGATTCTTTC | |
| eA3A-1F | AGAAAAAACCCCGGATTCTAGAACTAGTGGATCCCCCGGGAAAAAAATGGAAGCCAGCCCAGCATC | eA3A-BE3 |
| eA3A-1R | AAGATTCTTAGCCTGGCCGTGTAGAAAGCCCCTGTGC | |
| eA3A-2F | CAGGCTAAGAATCTTCTCTG | |
| eA3A-2R | ATTACTAAAGATCTCCTGCAGGTAGCAGATCCGATTCTTTC | |
| R128A-1F | AGAAAAAACCCCGGATTCTAGAACTAGTGGATCCCCCGGGAAAAAAATGAGTTCCGAGACAGGC | A3A(R128A)-BE3 |
| R128A-1R | TCGTAATCATAGATGGCGGCAGCGAAGATACG | |
| R128A-2F | CATCTATGATTACGACCCCC | |
| R128A-2R | ATTACTAAAGATCTCCTGCAGGTAGCAGATCCGATTCTTTC | |
| Y130F-1F | AGAAAAAACCCCGGATTCTAGAACTAGTGGATCCCCCGGGAAAAAAATGAGTTCCGAGACAGGC | A3A(Y130F)-BE3 |
| Y130F-1R | AGGGGGTCGTAATCAAAGATGCGGGCAGCGA | |
| Y130F-2F | TGATTACGACCCCCTATATA | |
| Y130F-2R | ATTACTAAAGATCTCCTGCAGGTAGCAGATCCGATTCTTTC | |

***Primers for amplification of target regions for high-throughput sequencing (HTS)***

| **Primer name** | **Sequence (5' → 3')** |
|---|---|
| PolyC-1-HTS-index1-F | ATCACGGGAAAGACTGGCTCATCAAAACC |
| PolyC-1-HTS-index1-R | GCCTAATTTTATAAATTTTGAGGAAGCG |
| PolyC-1-HTS-index2-F | CGATGTGGAAAGACTGGCTCATCAAAACC |
| PolyC-1-HTS-index2-R | TGGTCATTTTATAAATTTTGAGGAAGCG |
| PolyC-1-HTS-index3-F | AGTTCCGGAAAGACTGGCTCATCAAAACC |

| | |
|---|---|
| PolyC-1-HTS-index3-R | CTCTACTTTTATAAATTTTGAGGAAGCG |
| PolyC-1-HTS-index4-F | CACTCAGGAAAGACTGGCTCATCAAAACC |
| PolyC-1-HTS-index4-R | TGTTGGTTTTATAAATTTTGAGGAAGCG |
| PolyC-1-HTS-index5-F | GTGGCCGGAAAGACTGGCTCATCAAAACC |
| PolyC-1-HTS-index5-R | CGAAACTTTTATAAATTTTGAGGAAGCG |
| PolyC-1-HTS-index6-F | CGTACGGGAAAGACTGGCTCATCAAAACC |
| PolyC-1-HTS-index6-R | CCACTCTTTTATAAATTTTGAGGAAGCG |
| PolyC-1-HTS-index7-F | GGTAGCGGAAAGACTGGCTCATCAAAACC |
| PolyC-1-HTS-index7-R | ATCAGTTTTTATAAATTTTGAGGAAGCG |
| PolyC-2-HTS-index1-F | CACCGGTGCGCACATCAATCATTTTC |
| PolyC-2-HTS-index1-R | ATCGTGTGGCTTACTGTAAGCTACAGG |
| PolyC-2-HTS-index2-F | ATGAGCTGCGCACATCAATCATTTTC |
| PolyC-2-HTS-index2-R | AGGAATTGGCTTACTGTAAGCTACAGG |
| PolyC-2-HTS-index3-F | CAAAAGTGCGCACATCAATCATTTTC |
| PolyC-2-HTS-index3-R | TAGTTGTGGCTTACTGTAAGCTACAGG |
| PolyC-2-HTS-index4-F | TCGGCATGCGCACATCAATCATTTTC |
| PolyC-2-HTS-index4-R | GAATGATGGCTTACTGTAAGCTACAGG |
| PolyC-2-HTS-index5-F | TCCCGATGCGCACATCAATCATTTTC |
| PolyC-2-HTS-index5-R | CTTCGATGGCTTACTGTAAGCTACAGG |
| PolyC-2-HTS-index6-F | CTATACTGCGCACATCAATCATTTTC |
| PolyC-2-HTS-index6-R | TCTGAGTGGCTTACTGTAAGCTACAGG |
| PolyC-2-HTS-index7-F | TTAGGCTGCGCACATCAATCATTTTC |
| PolyC-2-HTS-index7-R | TGACCATGGCTTACTGTAAGCTACAGG |
| PolyC-3-HTS-index1-F | ACATGTTCCGCCATGTCCAACACG |
| PolyC-3-HTS-index1-R | CAGATCCTTTAATAATTTCCGAAATAGG |
| PolyC-3-HTS-index2-F | ACTTGATCCGCCATGTCCAACACG |
| PolyC-3-HTS-index2-R | GATCAGCTTTAATAATTTCCGAAATAGG |
| PolyC-3-HTS-index3-F | TAGCTTTCCGCCATGTCCAACACG |
| PolyC-3-HTS-index3-R | GGCTACCTTTAATAATTTCCGAAATAGG |
| PolyC-3-HTS-index4-F | CCGTCCTCCGCCATGTCCAACACG |
| PolyC-3-HTS-index4-R | GTAGAGCTTTAATAATTTCCGAAATAGG |
| PolyC-3-HTS-index5-F | GTCCGCTCCGCCATGTCCAACACG |
| PolyC-3-HTS-index5-R | GTGAAACTTTAATAATTTCCGAAATAGG |
| PolyC-3-HTS-index6-F | GTTTCGTCCGCCATGTCCAACACG |
| PolyC-3-HTS-index6-R | GAGTGGCTTTAATAATTTCCGAAATAGG |
| PolyC-3-HTS-index7-F | ACTGATTCCGCCATGTCCAACACG |
| PolyC-3-HTS-index7-R | ATTCCTCTTTAATAATTTCCGAAATAGG |
| PolyC-4-HTS-index1-F | CAACTAGGGAAAATAAAGGGAAAGACC |
| PolyC-4-HTS-index1-R | CACGATTGACCCAAAGGGAACATAAGAC |
| PolyC-4-HTS-index2-F | CAGGCGGGGAAAATAAAGGGAAAGACC |

| | |
|---|---|
| PolyC-4-HTS-index2-R | CATGGCTGACCCAAAGGGAACATAAGAC |
| PolyC-4-HTS-index3-F | ACAGTGGGGAAAATAAAGGGAAAGACC |
| PolyC-4-HTS-index3-R | GCCAATTGACCCAAAGGGAACATAAGAC |
| PolyC-4-HTS-index4-F | CTTGTAGGGAAAATAAAGGGAAAGACC |
| PolyC-4-HTS-index4-R | AGTCAATGACCCAAAGGGAACATAAGAC |
| PolyC-4-HTS-index5-F | ATGTCAGGGAAAATAAAGGGAAAGACC |
| PolyC-4-HTS-index5-R | CATTTTTGACCCAAAGGGAACATAAGAC |
| PolyC-4-HTS-index6-F | CCAACAGGGAAAATAAAGGGAAAGACC |
| PolyC-4-HTS-index6-R | CGGAATTGACCCAAAGGGAACATAAGAC |
| PolyC-4-HTS-index7-F | TCATTCGGGAAAATAAAGGGAAAGACC |
| PolyC-4-HTS-index7-R | CTAGCTTGACCCAAAGGGAACATAAGAC |
| PolyC-5-HTS-index1-F | CACCGGGTCACACCACGCTCAAACGG |
| PolyC-5-HTS-index1-R | ATCGTGAGATCTGGTCCACGGCCTGG |
| PolyC-5-HTS-index2-F | ATGAGCGTCACACCACGCTCAAACGG |
| PolyC-5-HTS-index2-R | AGGAATAGATCTGGTCCACGGCCTGG |
| PolyC-5-HTS-index3-F | CAAAAGGTCACACCACGCTCAAACGG |
| PolyC-5-HTS-index3-R | TAGTTGAGATCTGGTCCACGGCCTGG |
| PolyC-5-HTS-index4-F | TCGGCAGTCACACCACGCTCAAACGG |
| PolyC-5-HTS-index4-R | GAATGAAGATCTGGTCCACGGCCTGG |
| PolyC-5-HTS-index5-F | TCCCGAGTCACACCACGCTCAAACGG |
| PolyC-5-HTS-index5-R | CTTCGAAGATCTGGTCCACGGCCTGG |
| PolyC-5-HTS-index6-F | CTATACGTCACACCACGCTCAAACGG |
| PolyC-5-HTS-index6-R | TCTGAGAGATCTGGTCCACGGCCTGG |
| PolyC-5-HTS-index7-F | TTAGGCGTCACACCACGCTCAAACGG |
| PolyC-5-HTS-index7-R | TGACCAAGATCTGGTCCACGGCCTGG |
| PolyC-5-HTS-index8-F | ACATGTGTCACACCACGCTCAAACGG |
| PolyC-5-HTS-index8-R | CAGATCAGATCTGGTCCACGGCCTGG |
| PolyC-5-HTS-index9-F | ACTTGAGTCACACCACGCTCAAACGG |
| PolyC-5-HTS-index9-R | GATCAGAGATCTGGTCCACGGCCTGG |
| PolyC-5-HTS-index10-F | TAGCTTGTCACACCACGCTCAAACGG |
| PolyC-5-HTS-index10-R | GGCTACAGATCTGGTCCACGGCCTGG |
| PolyC-5-HTS-index11-F | CCGTCCGTCACACCACGCTCAAACGG |
| PolyC-5-HTS-index11-R | GTAGAGAGATCTGGTCCACGGCCTGG |
| PolyC-6-HTS-index1-F | GTCCGCTTCGGGGTTATTTATTTTTCG |
| PolyC-6-HTS-index1-R | GTGAAAGTTTGATTGGTCAAGTTGGC |
| PolyC-6-HTS-index2-F | GTTTCGTTCGGGGTTATTTATTTTTCG |
| PolyC-6-HTS-index2-R | GAGTGGGTTTGATTGGTCAAGTTGGC |
| PolyC-6-HTS-index3-F | ACTGATTTCGGGGTTATTTATTTTTCG |
| PolyC-6-HTS-index3-R | ATTCCTGTTTGATTGGTCAAGTTGGC |
| PolyC-6-HTS-index4-F | CAACTATTCGGGGTTATTTATTTTTCG |

| | |
|---|---|
| PolyC-6-HTS-index4-R | CACGATGTTTGATTGGTCAAGTTGGC |
| PolyC-6-HTS-index5-F | CAGGCGTTCGGGGTTATTTATTTTTCG |
| PolyC-6-HTS-index5-R | CATGGCGTTTGATTGGTCAAGTTGGC |
| PolyC-6-HTS-index6-F | ACAGTGTTCGGGGTTATTTATTTTTCG |
| PolyC-6-HTS-index6-R | GCCAATGTTTGATTGGTCAAGTTGGC |
| PolyC-6-HTS-index7-F | CTTGTATTCGGGGTTATTTATTTTTCG |
| PolyC-6-HTS-index7-R | AGTCAAGTTTGATTGGTCAAGTTGGC |
| PolyC-7-HTS-index1-F | CGATGTACTGCGGAAGTGAGGGGAGC |
| PolyC-7-HTS-index1-R | TGGTCATATCCGTGCGCGTAATCCTTCT |
| PolyC-7-HTS-index2-F | ATCACGACTGCGGAAGTGAGGGGAGC |
| PolyC-7-HTS-index2-R | GCCTAATATCCGTGCGCGTAATCCTTCT |
| PolyC-7-HTS-index3-F | AGTTCCACTGCGGAAGTGAGGGGAGC |
| PolyC-7-HTS-index3-R | CTCTACTATCCGTGCGCGTAATCCTTCT |
| PolyC-7-HTS-index4-F | CACTCAACTGCGGAAGTGAGGGGAGC |
| PolyC-7-HTS-index4-R | TGTTGGTATCCGTGCGCGTAATCCTTCT |
| PolyC-7-HTS-index5-F | GTGGCCACTGCGGAAGTGAGGGGAGC |
| PolyC-7-HTS-index5-R | CGAAACTATCCGTGCGCGTAATCCTTCT |
| PolyC-7-HTS-index6-F | CGTACGACTGCGGAAGTGAGGGGAGC |
| PolyC-7-HTS-index6-R | CCACTCTATCCGTGCGCGTAATCCTTCT |
| PolyC-7-HTS-index7-F | GGTAGCACTGCGGAAGTGAGGGGAGC |
| PolyC-7-HTS-index7-R | ATCAGTTATCCGTGCGCGTAATCCTTCT |
| PolyC-7-HTS-index8-F | CACCGGACTGCGGAAGTGAGGGGAGC |
| PolyC-7-HTS-index8-R | ATCGTGTATCCGTGCGCGTAATCCTTCT |
| PolyC-7-HTS-index9-F | ATGAGCACTGCGGAAGTGAGGGGAGC |
| PolyC-7-HTS-index9-R | AGGAATTATCCGTGCGCGTAATCCTTCT |
| PolyC-7-HTS-index10-F | CAAAAGACTGCGGAAGTGAGGGGAGC |
| PolyC-7-HTS-index10-R | TAGTTGTATCCGTGCGCGTAATCCTTCT |
| PolyC-7-HTS-index11-F | TCGGCAACTGCGGAAGTGAGGGGAGC |
| PolyC-7-HTS-index11-R | GAATGATATCCGTGCGCGTAATCCTTCT |
| PolyC-7-HTS-index12-F | TCCCGAACTGCGGAAGTGAGGGGAGC |
| PolyC-7-HTS-index12-R | CTTCGATATCCGTGCGCGTAATCCTTCT |
| PolyC-7-HTS-index13-F | CTATACACTGCGGAAGTGAGGGGAGC |
| PolyC-7-HTS-index13-R | TCTGAGTATCCGTGCGCGTAATCCTTCT |
| PolyC-7-HTS-index14-F | TTAGGCACTGCGGAAGTGAGGGGAGC |
| PolyC-7-HTS-index14-R | TGACCATATCCGTGCGCGTAATCCTTCT |
| PolyC-7-HTS-index15-F | ACATGTACTGCGGAAGTGAGGGGAGC |
| PolyC-7-HTS-index15-F | CAGATCTATCCGTGCGCGTAATCCTTCT |
| PolyC-7-HTS-index16-F | ACTTGAACTGCGGAAGTGAGGGGAGC |
| PolyC-7-HTS-index16-R | GATCAGTATCCGTGCGCGTAATCCTTCT |
| PolyC-7-HTS-index17-F | TAGCTTACTGCGGAAGTGAGGGGAGC |

| | |
|---|---|
| PolyC-7-HTS-index17-R | GGCTACTATCCGTGCGCGTAATCCTTCT |
| PolyC-7-HTS-index18-F | CCGTCCACTGCGGAAGTGAGGGGAGC |
| PolyC-7-HTS-index18-R | GTAGAGTATCCGTGCGCGTAATCCTTCT |
| PolyC-7-HTS-index19-F | GTCCGCACTGCGGAAGTGAGGGGAGC |
| PolyC-7-HTS-index19-R | GTGAAATATCCGTGCGCGTAATCCTTCT |
| PolyC-7-HTS-index20-F | GTTTCGACTGCGGAAGTGAGGGGAGC |
| PolyC-7-HTS-index20-R | GAGTGGTATCCGTGCGCGTAATCCTTCT |
| PolyC-7-HTS-index21-F | ACTGATACTGCGGAAGTGAGGGGAGC |
| PolyC-7-HTS-index21-R | ATTCCTTATCCGTGCGCGTAATCCTTCT |
| PolyC-7-HTS-index22-F | CAACTAACTGCGGAAGTGAGGGGAGC |
| PolyC-7-HTS-index22-R | CACGATTATCCGTGCGCGTAATCCTTCT |
| PolyC-7-HTS-index23-F | CAGGCGACTGCGGAAGTGAGGGGAGC |
| PolyC-7-HTS-index23-R | CATGGCTATCCGTGCGCGTAATCCTTCT |
| PolyC-7-HTS-index24-F | ACAGTGACTGCGGAAGTGAGGGGAGC |
| PolyC-7-HTS-index24-R | GCCAATTATCCGTGCGCGTAATCCTTCT |
| PolyC-8-HTS-index1-F | ATCACGTTCATCTATAAGGATATGGGTCG |
| PolyC-8-HTS-index1-R | CGATGTATAGCATATAATAAAAGTGGAG |
| PolyC-8-HTS-index2-F | TTAGGCTTCATCTATAAGGATATGGGTCG |
| PolyC-8-HTS-index2-R | TGACCAATAGCATATAATAAAAGTGGAG |
| PolyC-8-HTS-index3-F | ACAGTGTTCATCTATAAGGATATGGGTCG |
| PolyC-8-HTS-index3-R | GCCAATATAGCATATAATAAAAGTGGAG |
| PolyC-8-HTS-index4-F | CAGATCTTCATCTATAAGGATATGGGTCG |
| PolyC-8-HTS-index4-R | ACTTGAATAGCATATAATAAAAGTGGAG |
| PolyC-8-HTS-index5-F | GATCAGTTCATCTATAAGGATATGGGTCG |
| PolyC-8-HTS-index5-R | TAGCTTATAGCATATAATAAAAGTGGAG |
| PolyC-8-HTS-index6-F | GGCTACTTCATCTATAAGGATATGGGTCG |
| PolyC-8-HTS-index6-R | CTTGTAATAGCATATAATAAAAGTGGAG |
| PolyC-8-HTS-index7-F | AGTCAATTCATCTATAAGGATATGGGTCG |
| PolyC-8-HTS-index7-R | AGTTCCATAGCATATAATAAAAGTGGAG |
| PolyC-8-HTS-index8-F | ATGTCATTCATCTATAAGGATATGGGTCG |
| PolyC-8-HTS-index8-R | CCGTCCATAGCATATAATAAAAGTGGAG |
| PolyC-8-HTS-index9-F | GTAGAGTTCATCTATAAGGATATGGGTCG |
| PolyC-8-HTS-index9-R | GTCCGCATAGCATATAATAAAAGTGGAG |
| PolyC-8-HTS-index10-F | GTGAAATTCATCTATAAGGATATGGGTCG |
| PolyC-8-HTS-index10-R | GTGGCCATAGCATATAATAAAAGTGGAG |
| PolyC-8-HTS-index11-F | GTTTCGTTCATCTATAAGGATATGGGTCG |
| PolyC-8-HTS-index11-R | CGTACGATAGCATATAATAAAAGTGGAG |
| PolyC-8-HTS-index12-F | GAGTGGTTCATCTATAAGGATATGGGTCG |
| PolyC-8-HTS-index12-R | GGTAGCATAGCATATAATAAAAGTGGAG |
| PolyC-8-HTS-index13-F | ACTGATTTCATCTATAAGGATATGGGTCG |

| | |
|---|---|
| PolyC-8-HTS-index13-R | ATGAGCATAGCATATAATAAAAGTGGAG |
| PolyC-8-HTS-index14-F | ATTCCTTTCATCTATAAGGATATGGGTCG |
| PolyC-8-HTS-index14-R | CAAAAGATAGCATATAATAAAAGTGGAG |
| PolyC-8-HTS-index15-F | CAACTATTCATCTATAAGGATATGGGTCG |
| PolyC-8-HTS-index15-R | CACCGGATAGCATATAATAAAAGTGGAG |
| PolyC-8-HTS-index16-F | CACGATTTCATCTATAAGGATATGGGTCG |
| PolyC-8-HTS-index16-R | CACTCAATAGCATATAATAAAAGTGGAG |
| PolyC-8-HTS-index17-F | CAGGCGTTCATCTATAAGGATATGGGTCG |
| PolyC-8-HTS-index17-R | CATGGCATAGCATATAATAAAAGTGGAG |
| PolyC-8-HTS-index18-F | CATTTTTTCATCTATAAGGATATGGGTCG |
| PolyC-8-HTS-index18-R | CCAACAATAGCATATAATAAAAGTGGAG |
| PolyC-8-HTS-index19-F | CGGAATTTCATCTATAAGGATATGGGTCG |
| PolyC-8-HTS-index19-R | CTAGCTATAGCATATAATAAAAGTGGAG |
| PolyC-8-HTS-index20-F | CTATACTTCATCTATAAGGATATGGGTCG |
| PolyC-8-HTS-index20-R | CTCAGAATAGCATATAATAAAAGTGGAG |
| PolyC-8-HTS-index21-F | GACGACTTCATCTATAAGGATATGGGTCG |
| PolyC-8-HTS-index21-R | TAATCGATAGCATATAATAAAAGTGGAG |

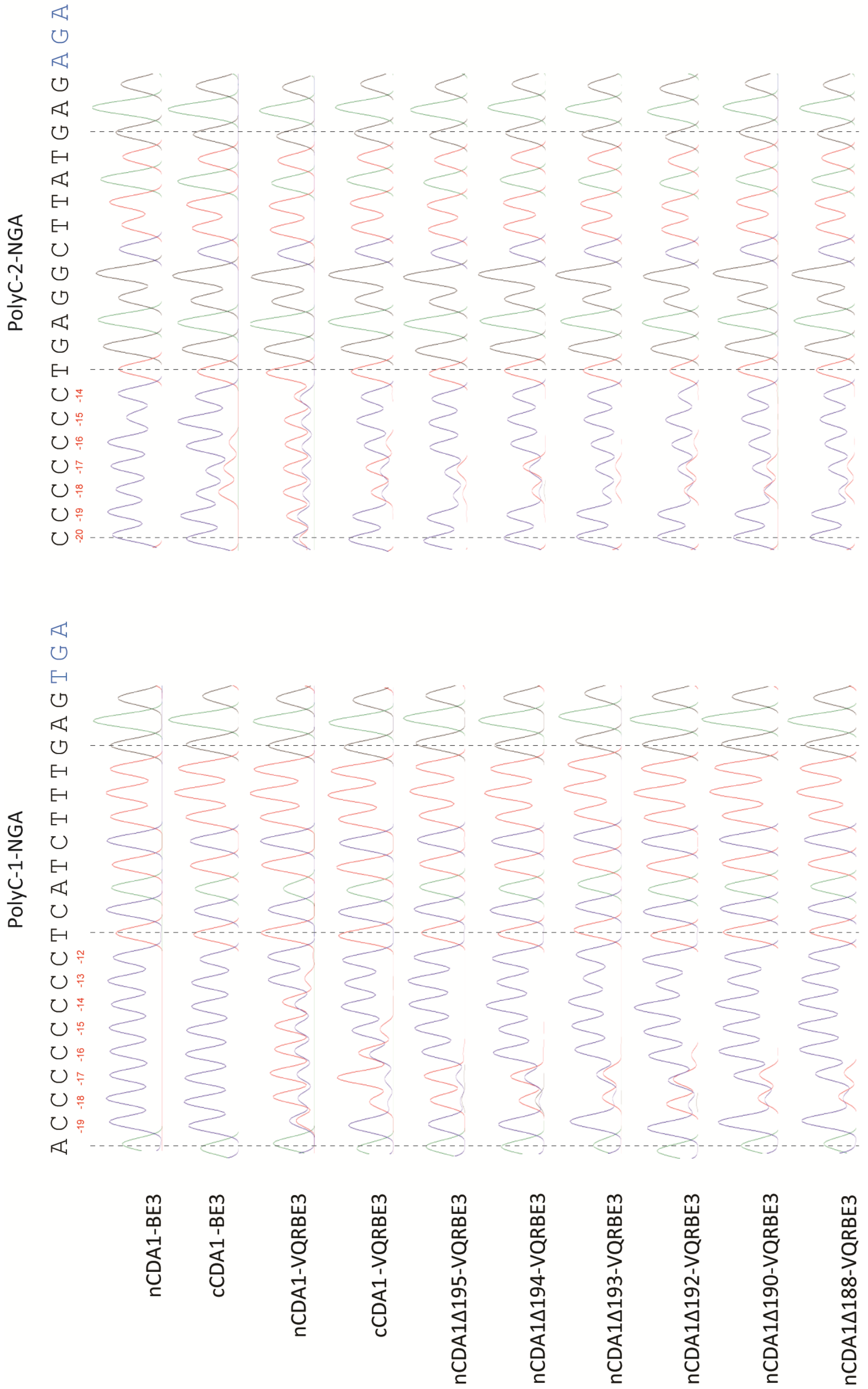


**Supplementary Figure 1** Analysis of base editing of a set of VQR-Cas9 BEs fused to the full-length CDA1 (nCDA1-VQRBE3) or C-terminally truncated versions of CDA1 (nCDA1Δ195-VQRBE3; nCDA1Δ194-VQRBE3; nCDA1Δ193-VQRBE3; nCDA1Δ192-VQRBE3; nCDA1Δ190-VQRBE3; nCDA1Δ188-VQRBE3; Fig. 1a,c). The two tested target sequences (PolyC-1-NGA and PolyC-2-NGA) both contain a polyC stretch upstream of the

PAM sequence NGA that is recognized by VQR-Cas9 (Fig. 1b). The amplified target sequences were directly sequenced by the dideoxy chain termination method (see Methods), and the sequence chromatograms are shown for each BE. The nucleotide sequences are shown above the chromatograms, with the PAM indicated in blue and the positions of the cytidines in the polyC stretch given in red (relative to the PAM). The reciprocal full-length fusion, cCDA1-VQRBE3, was also analyzed. nCDA1-BE3 and cCDA1-BE3 have no PAM in proper distance from the polyC stretch and served as negative controls.

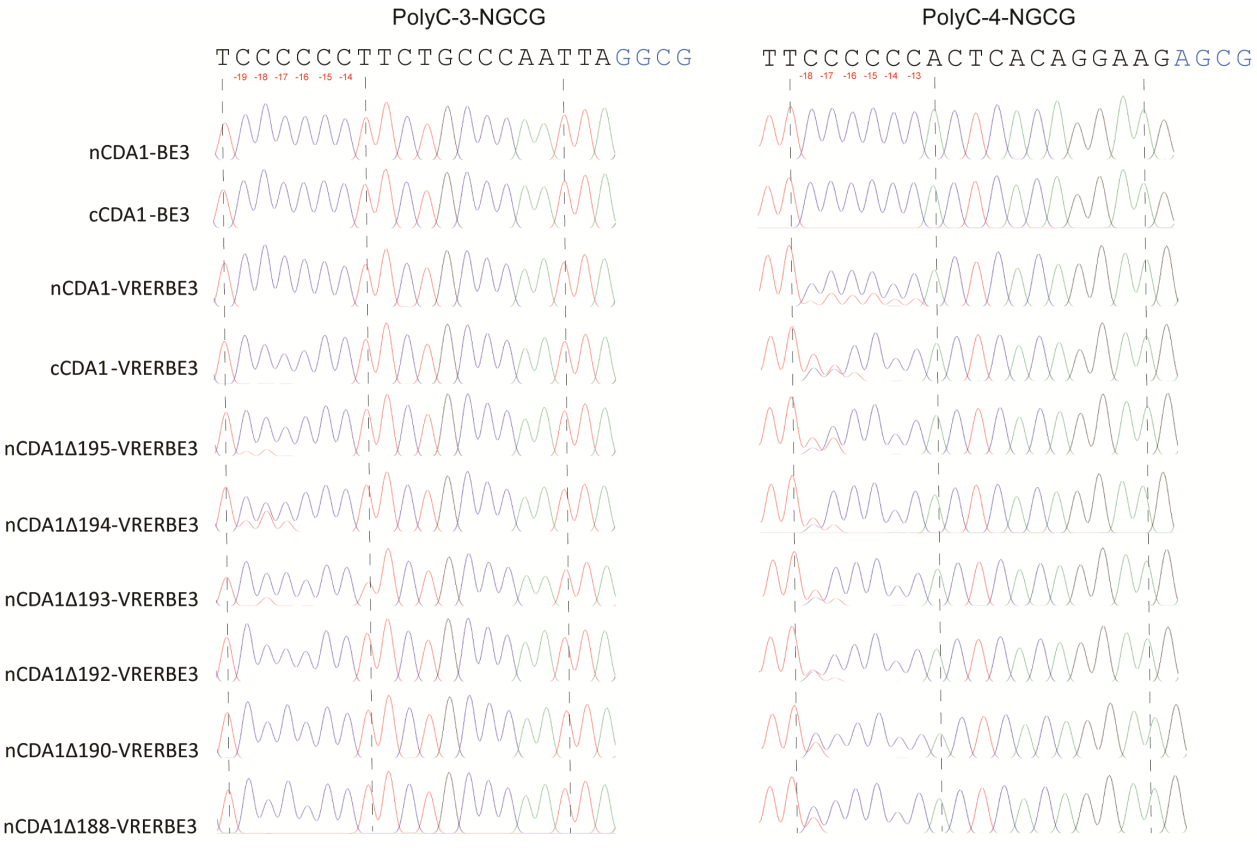


**Supplementary Figure 2** Analysis of base editing of a set of VRER-Cas9 BEs fused to the full-length CDA1 (nCDA1-VRERBE3) or C-terminally truncated versions of CDA1 (nCDA1Δ195-VRERBE3; nCDA1Δ194-VRERBE3; nCDA1Δ193-VRERBE3; nCDA1Δ192-VRERBE3; nCDA1Δ190-VRERBE3; nCDA1Δ188-VRERBE3; Fig. 1a,d). The two tested target sequences (PolyC-3-NGCG and PolyC-4-NGCG) both contain a polyC stretch upstream of the PAM sequence NGCG that is recognized by VRER-Cas9 (Fig. 1b). The amplified target sequences were directly sequenced by the dideoxy chain termination method (see Methods), and the sequence chromatograms are shown for each BE. The nucleotide sequences are shown above the chromatograms, with the PAM indicated in blue and the positions of the cytidines in the polyC stretch given in red (relative to the PAM). The reciprocal full-length fusion, cCDA1-VRERBE3, was also analyzed. nCDA1-BE3 and cCDA1-BE3 have no PAM in proper distance from the polyC stretch and served as negative controls.

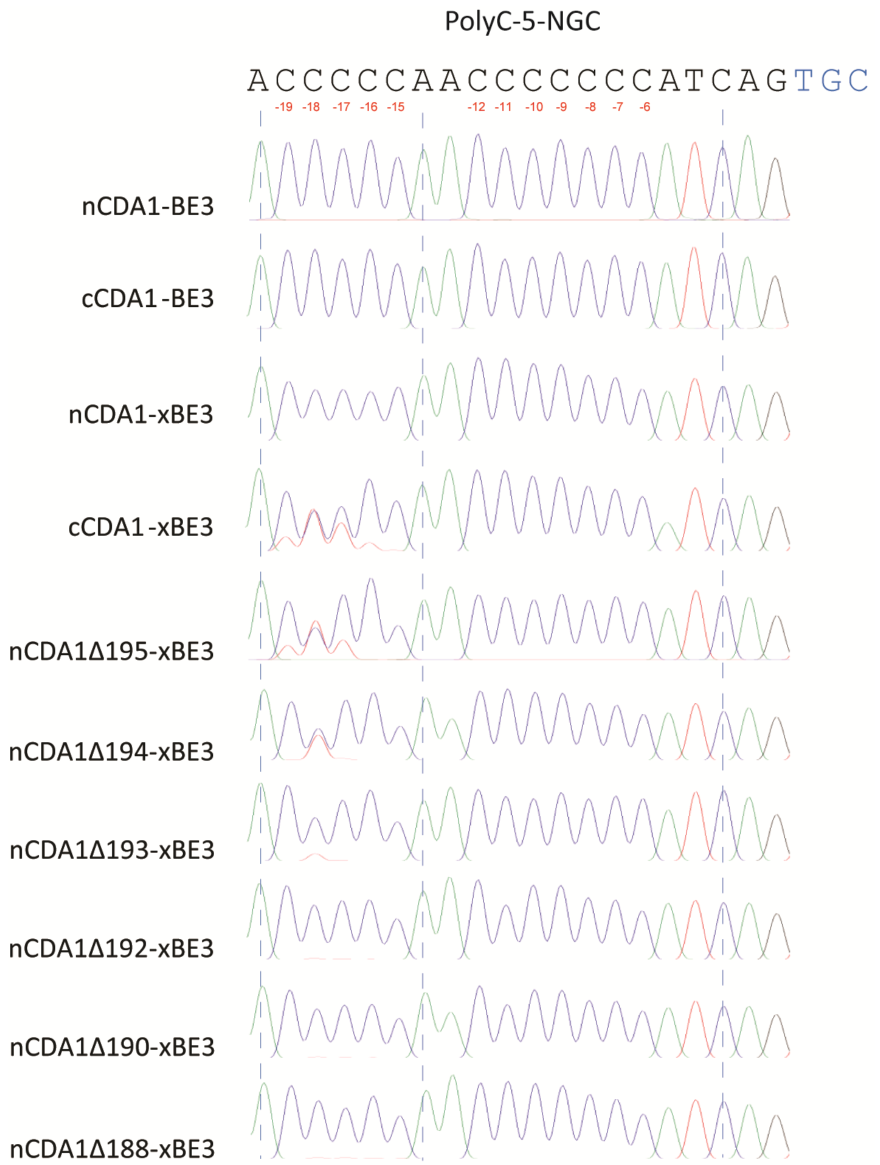


**Supplementary Figure 3** Analysis of base editing of a set of xCas9 BEs fused to the full-length CDA1 (nCDA1-xBE3) or C-terminally truncated versions of CDA1 (nCDA1Δ195-xBE3; nCDA1Δ194-xBE3; nCDA1Δ193-xBE3; nCDA1Δ192-xBE3; nCDA1Δ190-xBE3; nCDA1Δ188-xBE3; Fig. 1a,e). The tested target sequence (PolyC-5-NGC) contains a polyC stretch upstream of the PAM sequence NG that is recognized by xCas9 (Fig. 1b). The amplified target sequences were directly sequenced by the dideoxy chain termination method (see Methods), and the sequence chromatograms are shown for each BE. The nucleotide sequence is shown above the chromatograms, with the PAM indicated in blue and the positions of the cytidines in the polyC stretch given in red (relative to the PAM). The

reciprocal full-length fusion, cCDA1-xBE3, was also analyzed. nCDA1-BE3 and cCDA1-BE3 have no PAM in proper distance from the polyC stretch and served as negative controls.

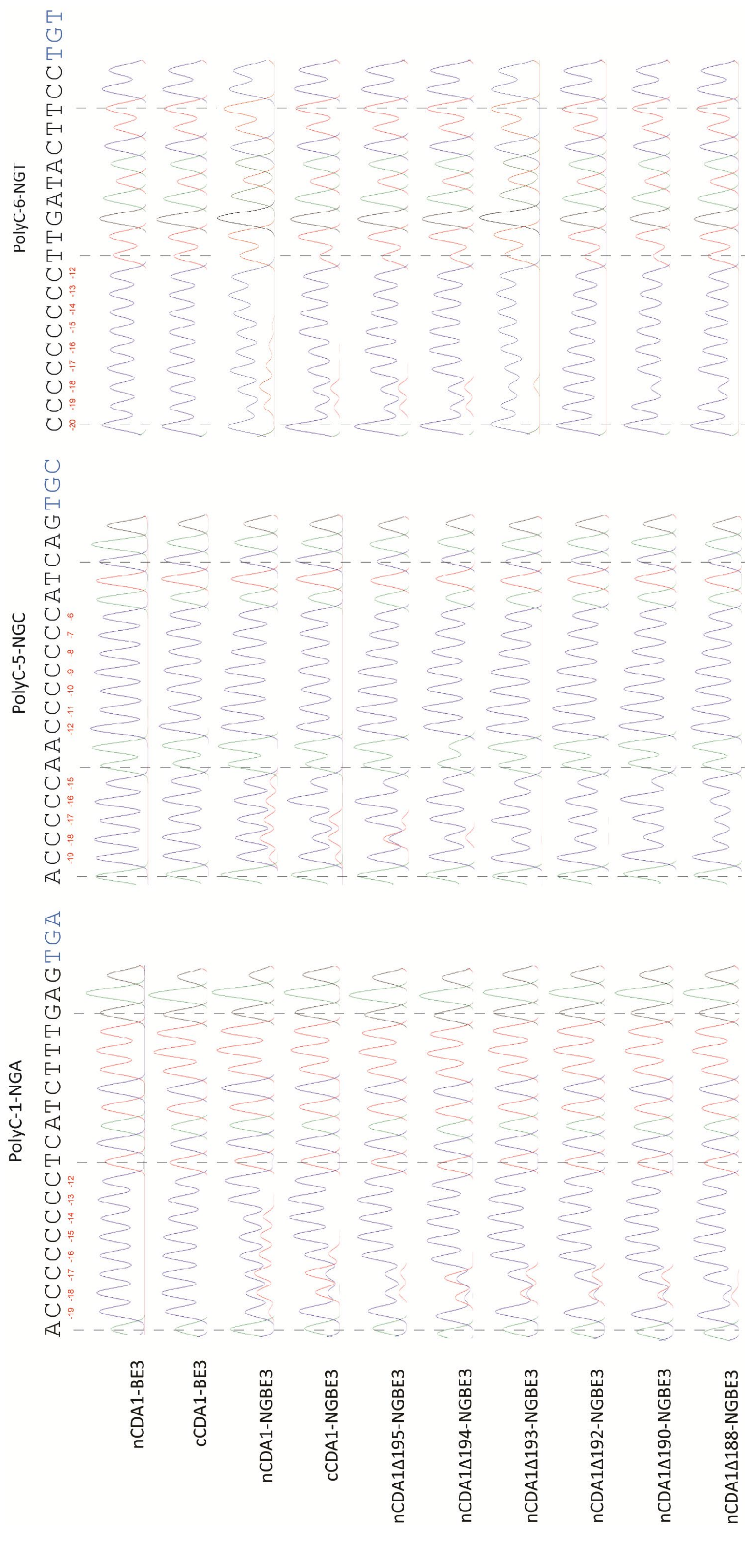


**Supplementary Figure 4** Analysis of base editing of a set of SpCas9-NG BEs fused to the full-length CDA1 (nCDA1-NGBE3) or C-terminally truncated versions of CDA1 (nCDA1Δ195-NGBE3; nCDA1Δ194-NGBE3; nCDA1Δ193-NGBE3; nCDA1Δ192-NGBE3;

nCDA1Δ190-NGBE3; nCDA1Δ188-NGBE3; Fig. 1a,f,g). The three tested target sequences (PolyC-1-NGA, PolyC-5-NGC and PolyC-6-NGT) all contain a polyC stretch upstream of the PAM sequence NG that is recognized by SpCas9-NG (Fig. 1b). The amplified target sequences were directly sequenced by the dideoxy chain termination method (see Methods), and the sequence chromatograms are shown for each BE. The nucleotide sequences are shown above the chromatograms, with the PAM indicated in blue and the positions of the cytidines in the polyC stretch given in red (relative to the PAM). The reciprocal full-length fusion, cCDA1-NGBE3, was also analyzed. nCDA1-BE3 and cCDA1-BE3 have no PAM in proper distance from the polyC stretch and served as negative controls.

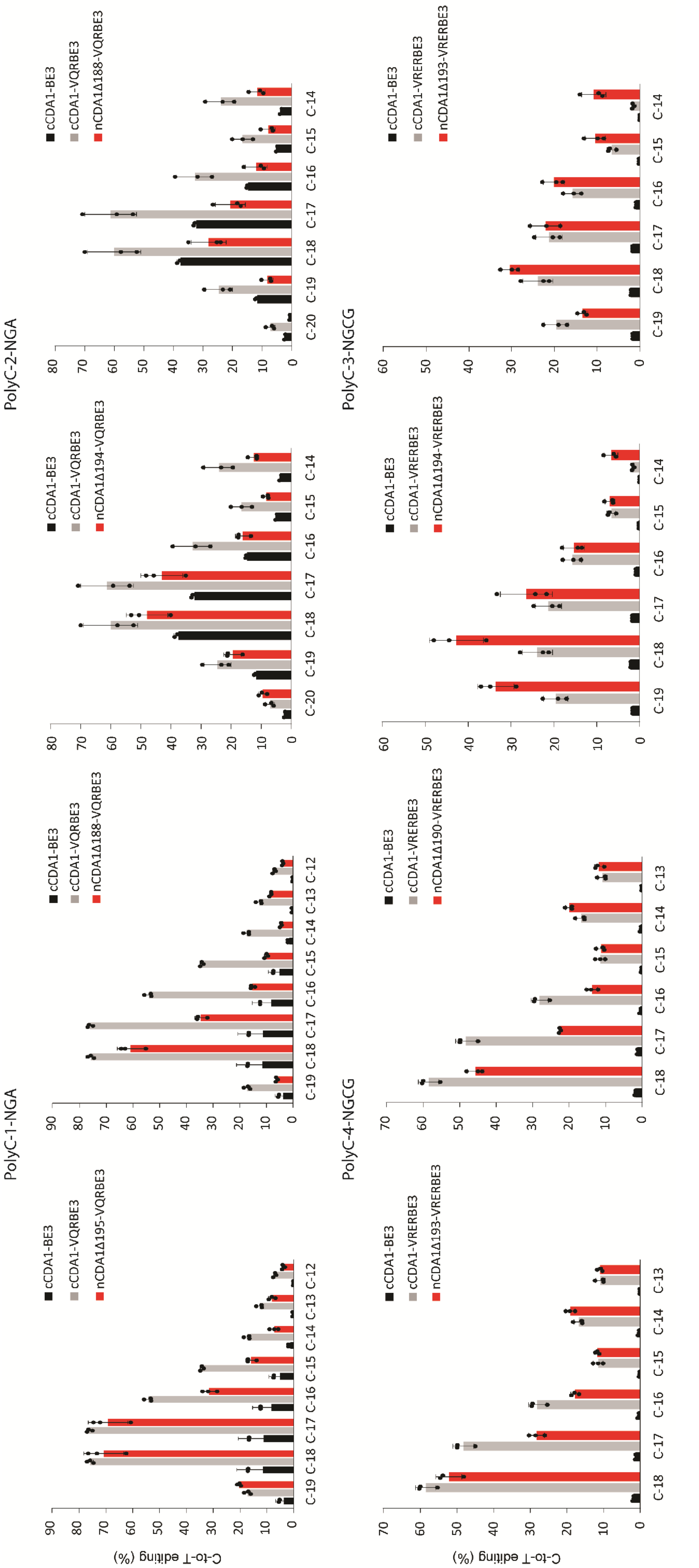
PolyC-1-NGA
PolyC-2-NGA
PolyC-4-NGCG
PolyC-3-NGCG
C-to-T editing (%)
cCDA1-BE3
cCDA1-VQRBE3
nCDA1Δ195-VQRBE3
nCDA1Δ188-VQRBE3
nCDA1Δ194-VQRBE3
cCDA1-VRERBE3
nCDA1Δ193-VRERBE3
nCDA1Δ190-VRERBE3
C-20 C-19 C-18 C-17 C-16 C-15 C-14 C-13 C-12

**Supplementary Figure 5** Base editing outcomes of cCDA1-VQRBE3, cCDA1-VRERBE3 and BE variants with SpCas9-VQR or SpCas9-VRER fusions to C-terminally truncated CDA1 variants. Two NGA target sites were tested (PolyC-1-NGA, PolyC-2-NGA; for sequences see Supplementary Table 1; for sequence chromatograms, see Supplementary Figure 1) for BEs with SpCas9-VQR and two NGCG target sites were tested (PolyC-3-NGCG, PolyC-4-NGCG; for sequences see Supplementary Table 1; for sequence chromatograms, see Supplementary Figure 2) for BEs with SpCas9-VRER. For comparison, the cCDA1-BE3 editor (recognizing the PAM sequence NGG) was also included. % of C-to-T editing represents the percentage of total sequencing reads with the target C converted to T. Values and error bars represent the mean and standard deviation of three independent biological replicates. Source data are provided as a Source Data file.

PolyC-1-NGA

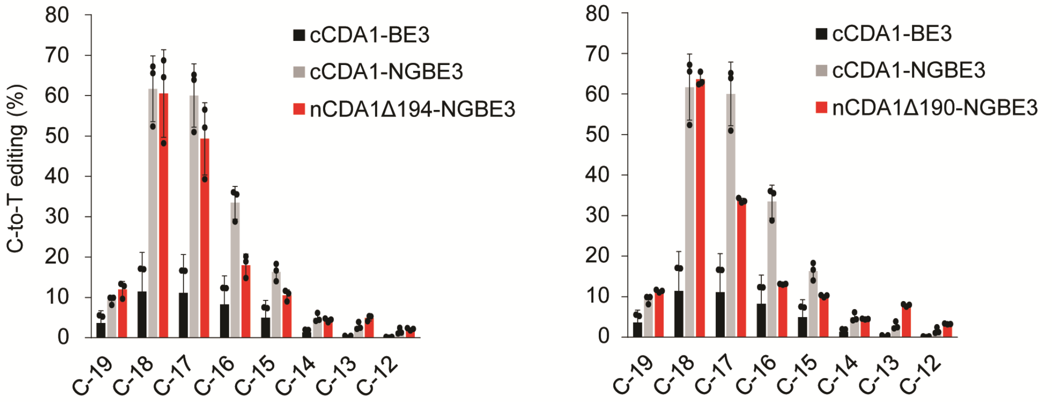


PolyC-5-NGC

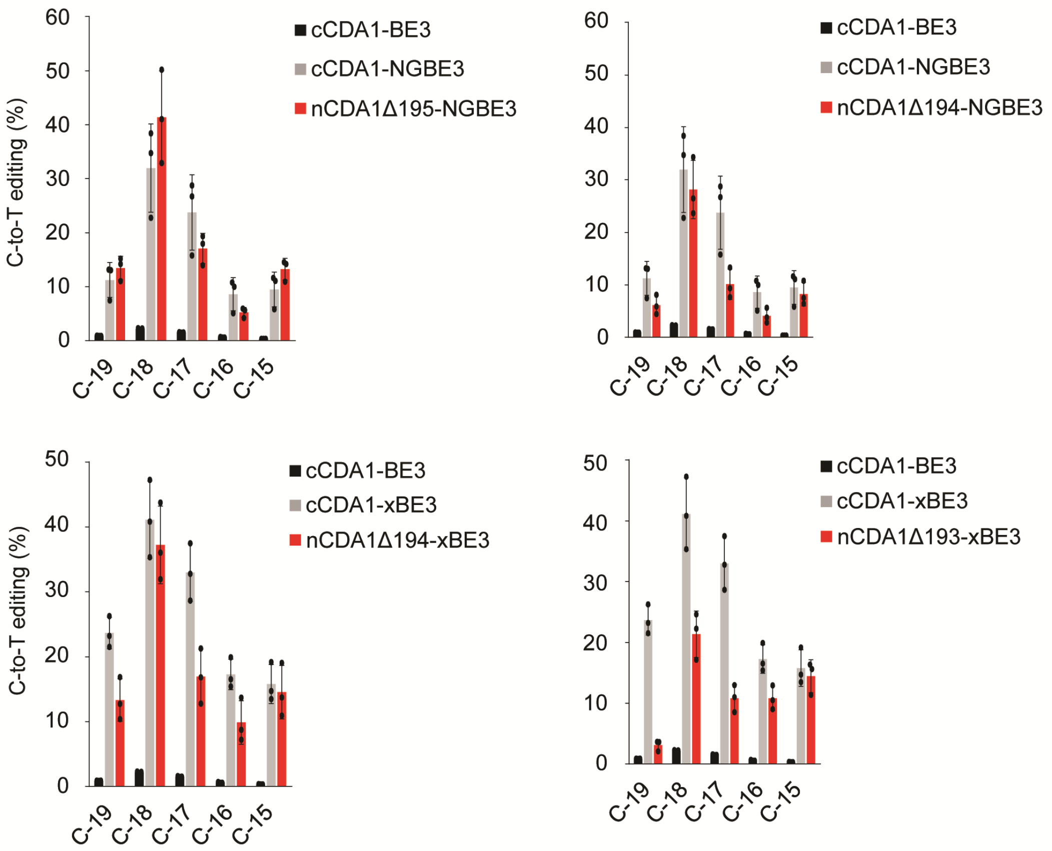


PolyC-6-NGT

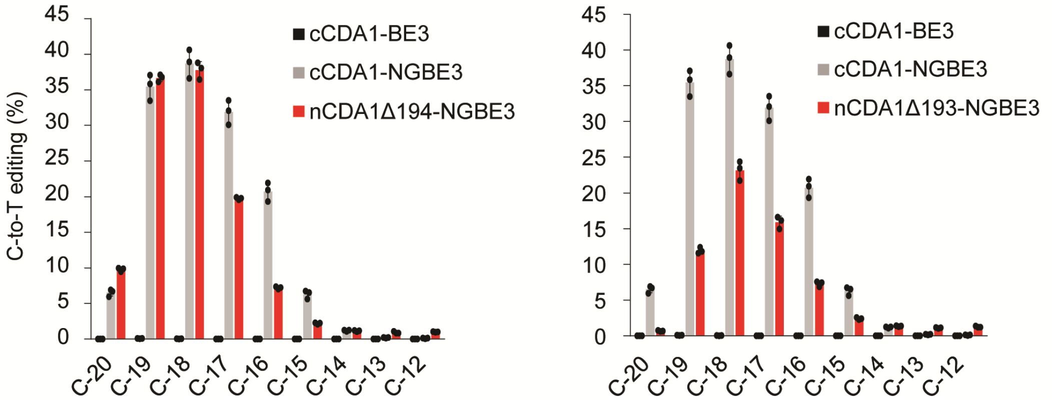

**Supplementary Figure 6** Base editing outcomes of cCDA1-NGBE3, cCDA1-xBE3 and BE variants with SpCas9-NG or xCas9 fusions to C-terminally truncated CDA1 variants. Three non-NGG target sites were tested (PolyC-1-NGA, PolyC-5-NGC and PolyC-6-NGT; for sequences see Supplementary Table 1; for sequence chromatograms, see Supplementary Figures 3 and 4). For comparison the cCDA1-BE3 editor (recognizing the PAM sequence NGG) was also included. % of C-to-T editing represents the percentage of total sequencing reads with the target C converted to T. Values and error bars represent the mean and standard deviation of three independent biological replicates. Source data are provided as a Source Data file.

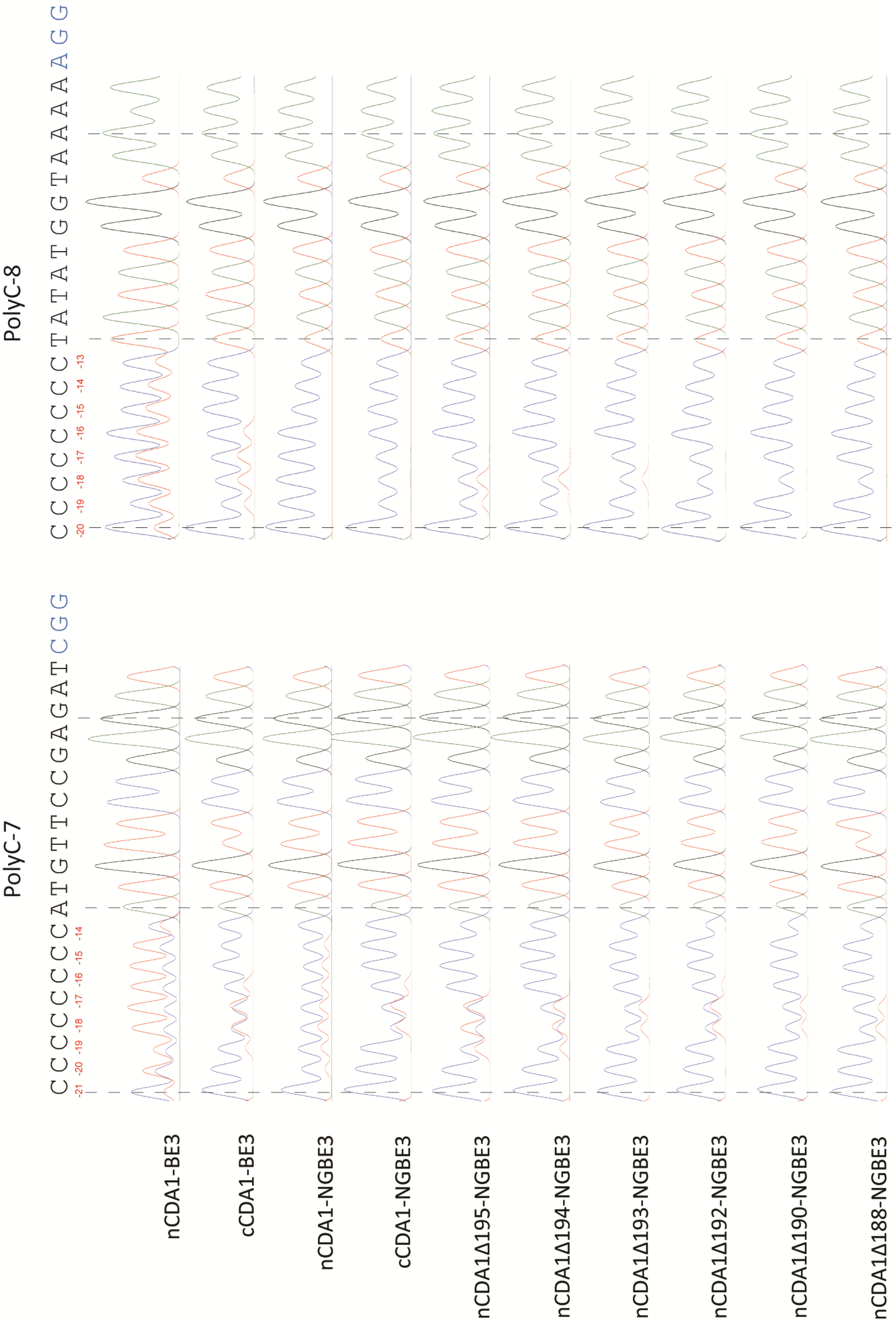


**Supplementary Figure 7** Analysis of base editing of a set of SpCas9-NG BEs fused to the full-length CDA1 (nCDA1-NGBE3) or C-terminally truncated versions of CDA1

(nCDA1Δ195-NGBE3; nCDA1Δ194-NGBE3; nCDA1Δ193-NGBE3; nCDA1Δ192-NGBE3; nCDA1Δ190-NGBE3; nCDA1Δ188-NGBE3) in two target sequences containing the PAM sequence NGG (PolyC-7, PolyC-8). Both target sequences contain a polyC stretch upstream of the PAM. Note that the PAM sequence NGG can be recognized by both the wild-type Cas9 (as in nCDA1-BE3 and cCDA1-BE3) and the SpCas9-NG variants. The amplified target sequences were directly sequenced by the dideoxy chain termination method (see Methods), and the sequence chromatograms are shown for each BE. The nucleotide sequences are shown above the chromatograms, with the PAM indicated in blue and the positions of the cytidines in the polyC stretch given in red (relative to the PAM). The reciprocal full-length fusion, cCDA1-NGBE3, was also analyzed.

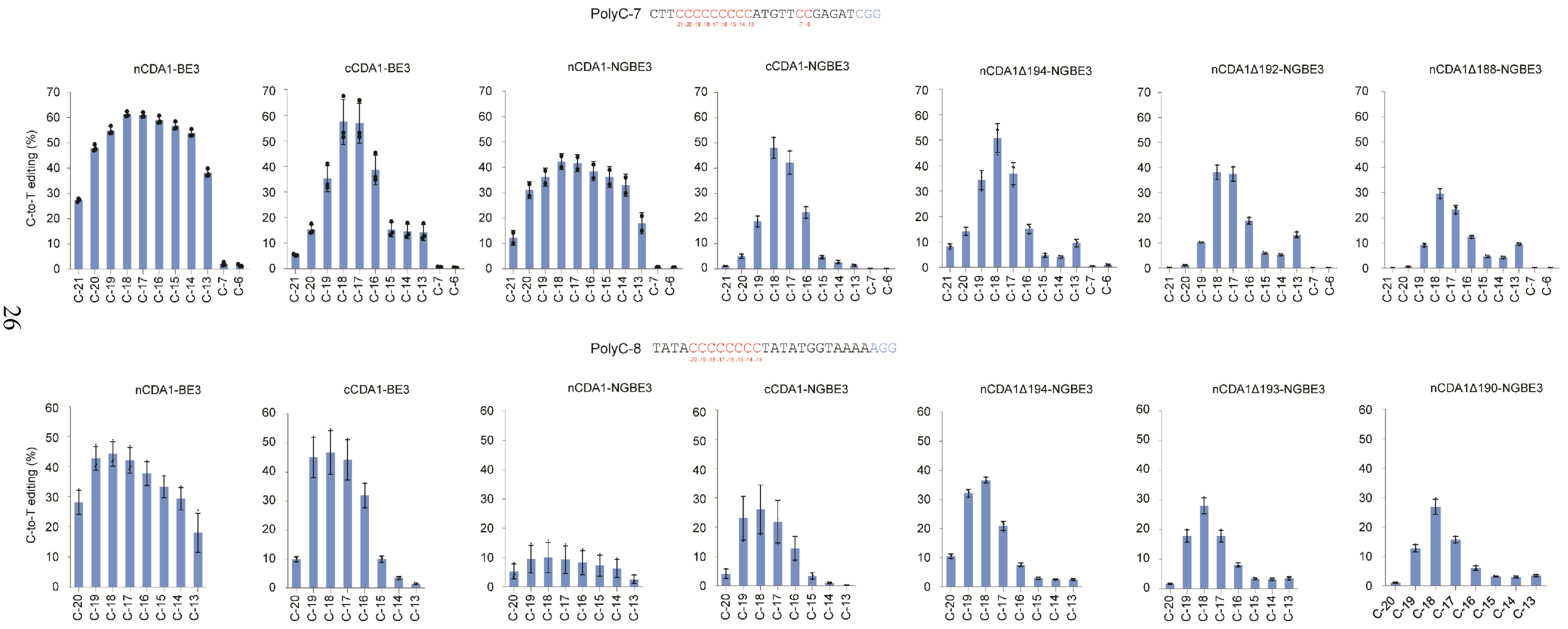
PolyC-7 CTTCCCCCCCCCATGTTCCGAGATCGG
-21 -20 -19 -18 -17 -16 -15 -14 -13 -7 -6
nCDA1-BE3
cCDA1-BE3
nCDA1-NGBE3
cCDA1-NGBE3
nCDA1Δ194-NGBE3
nCDA1Δ192-NGBE3
nCDA1Δ188-NGBE3
C-to-T editing (%)
C-21 C-20 C-19 C-18 C-17 C-16 C-15 C-14 C-13 C-7 C-6
PolyC-8 TATACCCCCCCCTATATGGTAAAAAGG
-20 -19 -18 -17 -16 -15 -14 -13
nCDA1-BE3
cCDA1-BE3
nCDA1-NGBE3
cCDA1-NGBE3
nCDA1Δ194-NGBE3
nCDA1Δ193-NGBE3
nCDA1Δ190-NGBE3
C-to-T editing (%)
C-20 C-19 C-18 C-17 C-16 C-15 C-14 C-13

**Supplementary Figure 8** Quantitative analysis of base editing outcomes obtained with a set of SpCas9-NG BEs fused to the full-length CDA1 (nCDA1-NGBE3) or C-terminally truncated versions of CDA1 (nCDA1Δ194-NGBE3; nCDA1Δ193-NGBE3; nCDA1Δ192-NGBE3; nCDA1Δ190-NGBE3; nCDA1Δ188-NGBE3) in comparison to the two full-length CDA1 fusions to the wild-type Cas9 (nCDA1-BE3; cCDA1-BE3). Both tested target sequences contain the PAM sequence NGG (PolyC-7, PolyC-8). The nucleotide sequences are shown above the diagrams, with the PAM indicated in blue and the positions of the cytidines in the polyC stretch given in red (relative to the PAM). % of C-to-T editing represents the percentage of total sequencing reads with the target C converted to T. Values and error bars represent the mean and standard deviation of three independent biological replicates. Source data are provided as a Source Data file.

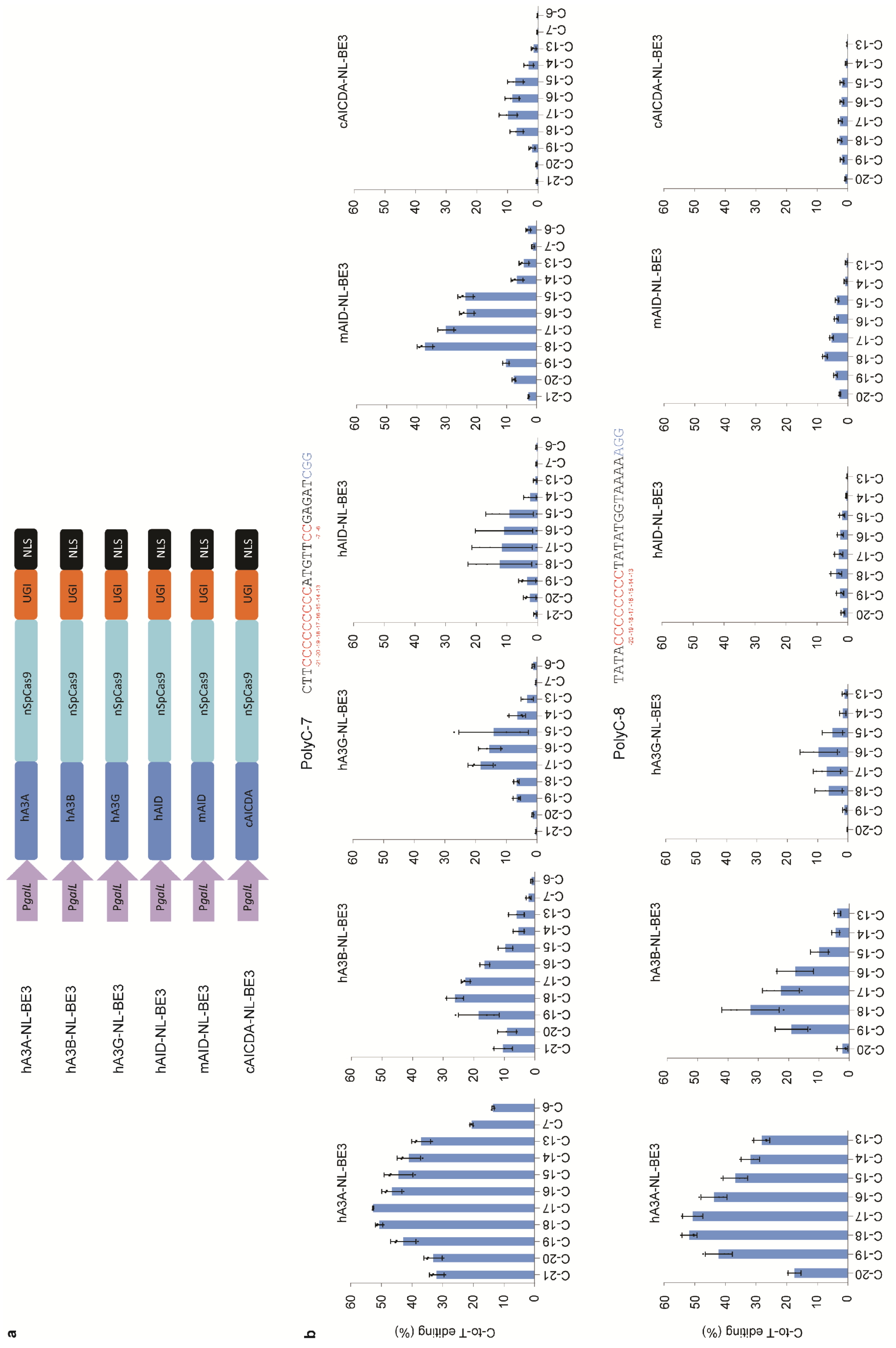

a
hA3A-NL-BE3
hA3B-NL-BE3
hA3G-NL-BE3
hAID-NL-BE3
mAID-NL-BE3
cAICDA-NL-BE3
PgalL
hA3A
hA3B
hA3G
hAID
mAID
cAICDA
nSpCas9
UGI
NLS
b
PolyC-7
CTTCCCCCCCCATGTTCCGAGATCGG
-21 -20 -19 -18 -17 -16 -15 -14 -13
-7 -6
PolyC-8
TATACCCCCCCCTATATGGTAAAAAGG
-20 -19 -18 -17 -16 -15 -14 -13
C-to-T editing (%)

**Supplementary Figure 9** Screening of deaminases directly fused to Cas9 (without linkers) for efficient base editing. **a** Series of new BEs constructed by directly fusing different APOBEC and AID deaminases with the Cas9 nickase (nCas9) and the uracil DNA glycosylase inhibitor (UGI) when targeting two polyC sites (polyC-7 and polyC-8). **b** Base editing outcomes of new BEs when targeting two polyC sites (polyC-7 and polyC-8). The sequence of each target site is shown with the numbers indicating the position of possible editing targets (red) relative to the PAM (blue). % of C-to-T editing represents the percentage of total sequencing reads with the target C converted to T. Values and error bars represent the mean and standard deviation of three independent biological replicates. hA3A: human *APOBEC3A* (GenBank accession number NM_145699); hA3B: human *APOBEC3B* (NM_004900); hA3G: human *APOBEC3G* (NM_021822); hAID: human *AID* (NM_020661); mAID: mouse *AID* (NM_009645); cAICDA: channel catfish *AICDA* (NM_001200185). Source data underlying panels **b** are provided as a Source Data file.

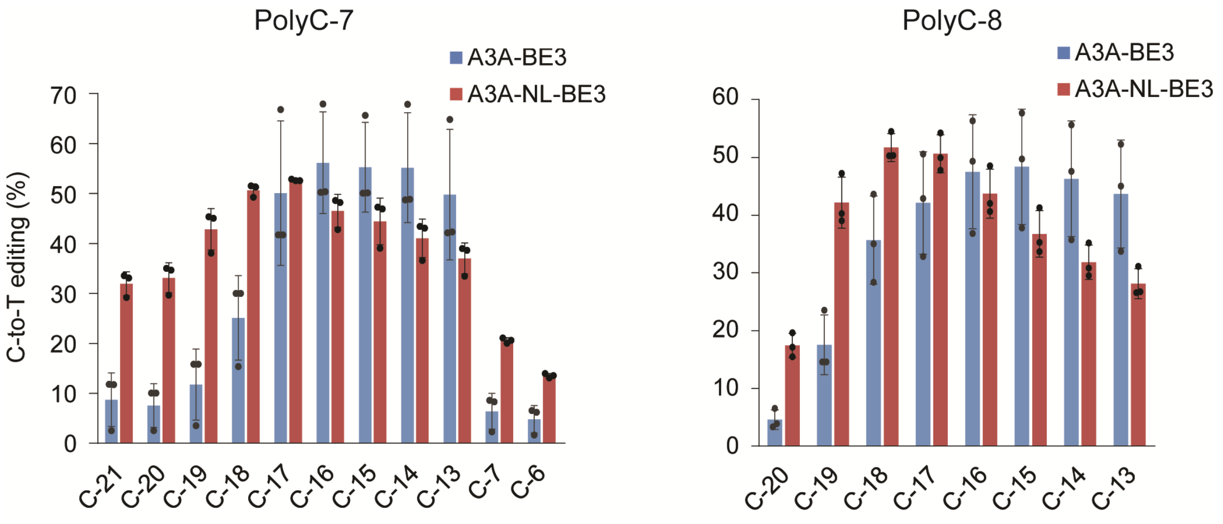


**Supplementary Figure 10** Removal of the linker between A3A and nCas9 broadens the width of the editing window while not appreciably affecting editing efficiency. Two polyC target sequences (polyC-7 and polyC-8; see Supplementary Figure 9) were tested. The x-axis shows the target Cs and their positions relative to the PAM. % of C-to-T editing represents the percentage of total sequencing reads with the target C converted to T. Values and error bars represent the mean and standard deviation of three independent biological replicates. Source data are provided as a Source Data file.

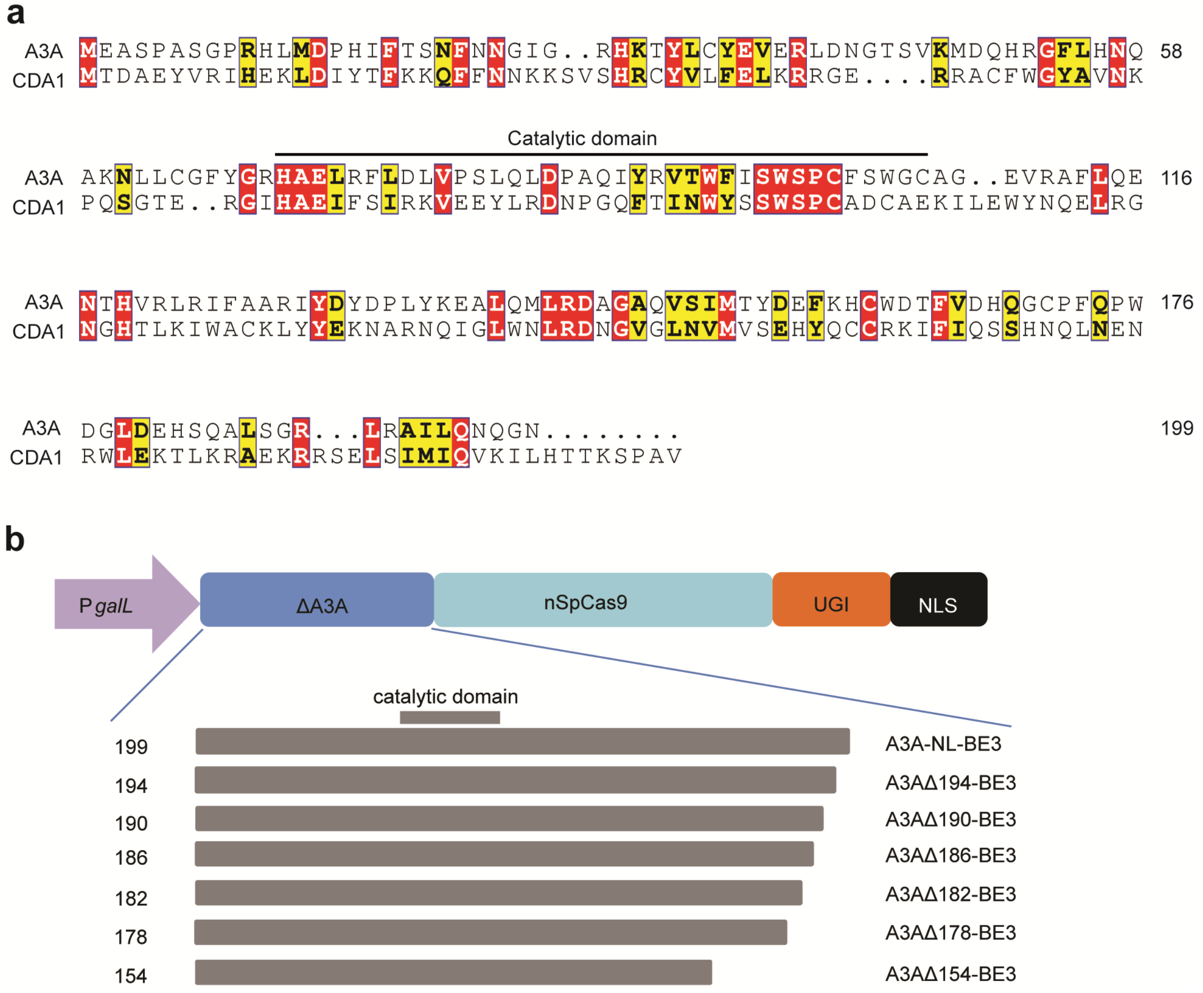


**Supplementary Figure 11** Design of base editors with truncated A3A domains. **a** Amino acid sequence alignment of A3A and CDA1. The catalytic domain is indicated by the black horizontal line. The alignment was created by CLUSTAL W (ref. 43; https://www.genome.jp/tools-bin/clustalw) and graphically formatted with the help of the ESPript 3.0 server (ref. 44; http://espript.ibcp.fr/ESPript/ESPript/). Identical amino acid residues are marked in red, similar residues in yellow. **b** Schematic representation of BEs with C-terminal A3A truncations. The truncated variants are named after the last A3A residue included.

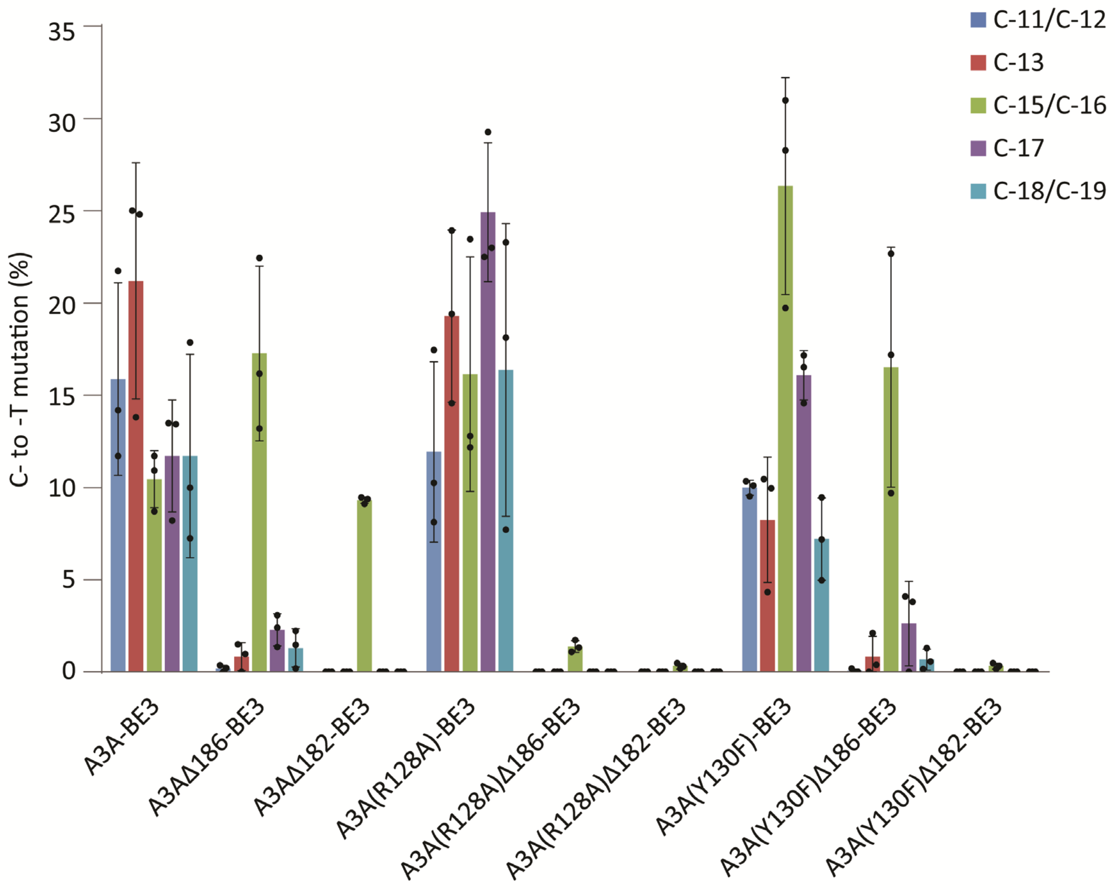


**Supplementary Figure 12** Base editing outcomes of A3A-BE3, two narrow-window A3AΔ-BE3 variants, two recently reported mutant BE3 variants that reduce off-target RNA editing (A3A(R128A)-BE3, A3A(Y130F)-BE3; 33) and the combinations of these mutations with each of the two narrow-window A3AΔ-BE3 variants. Five sites in the yeast *Can1* gene (containing Cs at different positions) were targeted and their sequences are listed in Supplementary Table 1. Edited clones were identified by using the canavanine selection strategy (see Methods). The x-axis represents the target Cs within the protospacers. The y-axis shows their C-to-T editing frequencies (see Methods). Values and error bars represent the mean and standard deviation of three independent biological replicates. Source data are provided as a Source Data file.

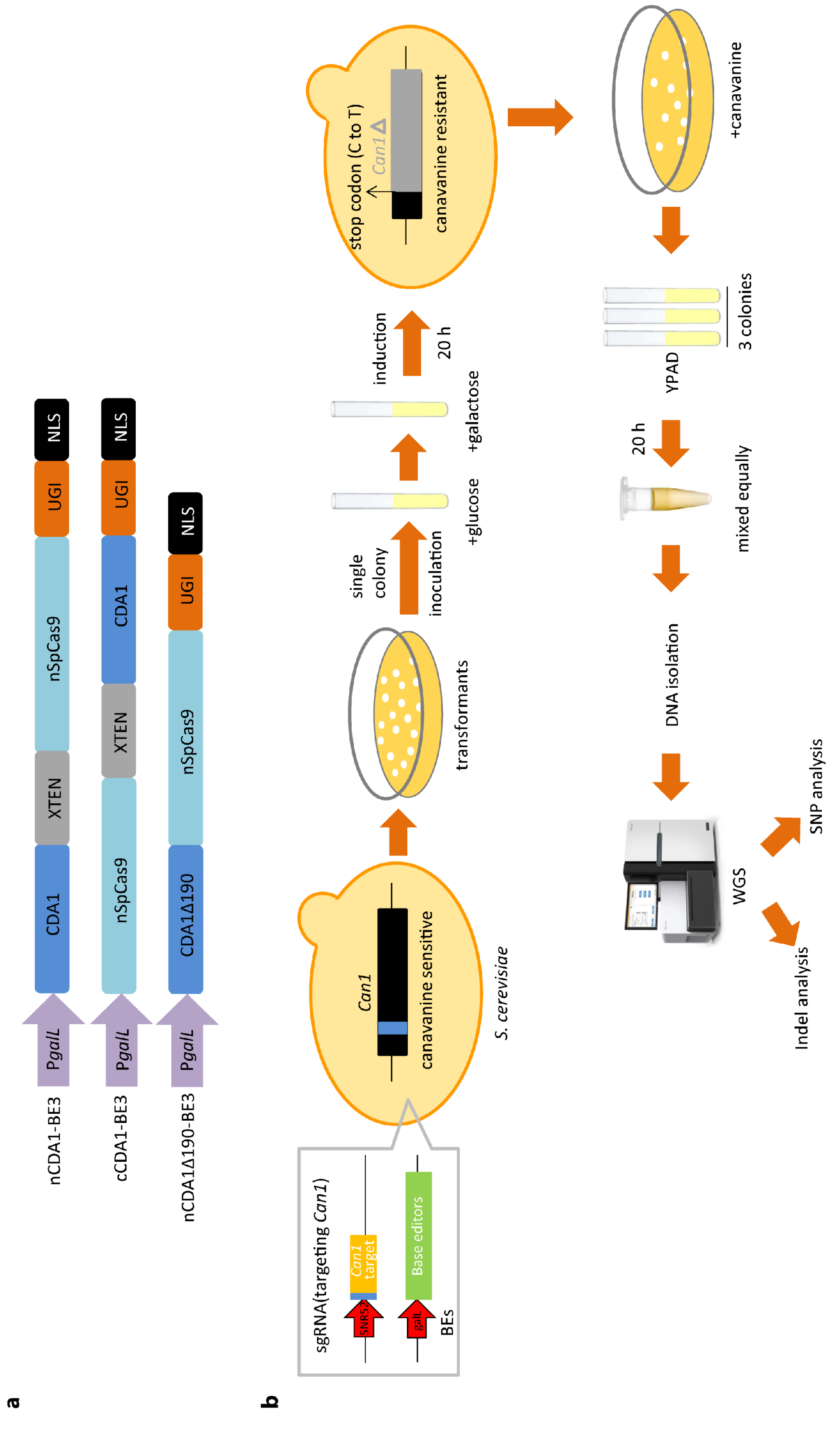
a
nCDA1-BE3
cCDA1-BE3
nCDA1Δ190-BE3
PgalL
CDA1
XTEN
nSpCas9
UGI
NLS
CDA1Δ190
b
sgRNA(targeting Can1)
SNR52
Can1 target
gall
Base editors
BEs
Can1
canavanine sensitive
S. cerevisiae
transformants
single colony
inoculation
+glucose
+galactose
induction
20 h
stop codon (C to T)
Can1Δ
canavanine resistant
+canavanine
YPAD
3 colonies
20 h
mixed equally
DNA isolation
WGS
Indel analysis
SNP analysis

**Supplementary Figure 13** Analysis of off-target editing induced by base editors nCDA1-BE3, cCDA1-BE3 and nCDA1Δ190-BE3 in yeast. **a** Schematic representation of the three base editors. **b** Experimental workflow.

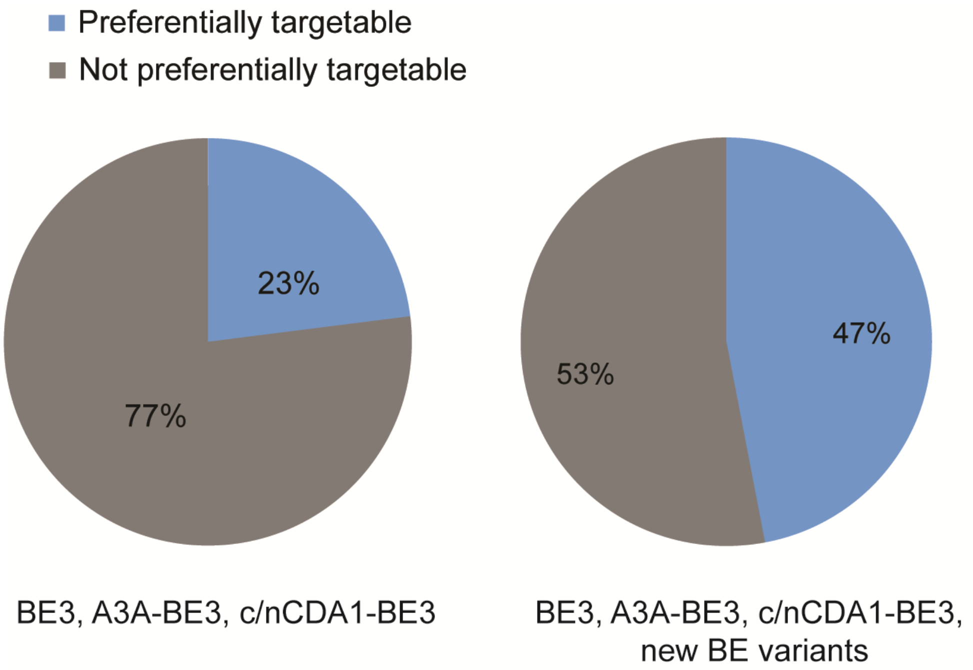


**Supplementary Figure 14** Fraction of pathogenic T-to-C and A-to-G SNPs in the ClinVar database (5074 mutations in total; ref. 8) that can be precisely addressed by the BEs available previously (left) and the expanded set of BEs that includes the high-precision BEs developed in the course of this work (right). The editing windows were assumed as follows: BE3 and A3A-BE3: $C_{-13}$ to $C_{-17}$; cCDA1-BE3: $C_{-16}$ to $C_{-19}$; nCDA1-BE3: $C_{-14}$ to $C_{-20}$ (refs. 15, 22, 23). The BE variants described in this work, including the A3AΔ-BE3 and nCDA1Δ-BE3 mutants, were assumed to have a narrowed window ($C_{-15}$ to $C_{-16}$) recognizing the PAM NGG and an editing preference of $C_{-18} > C_{-17} > C_{-19}$ recognizing the PAM NG, respectively.
